# High Performance Shape-Stabilized Phase Change Material with Nanoflower-Like Wrinkled Mesoporous Silica Encapsulating Polyethylene Glycol: Preparation and Thermal Properties

**DOI:** 10.3390/nano8060385

**Published:** 2018-05-31

**Authors:** Junkai Gao, Wenwen Tao, Dian Chen, Xiuwang Guo, Yan Chen, Yanjun Jiang

**Affiliations:** 1 School of Port and Transportation Engineering, Zhejiang Ocean University, Zhoushan 316022, China; gaojk@zjou.edu.cn (J.G.); 18868005586@163.com (W.T.); chendian0513@163.com (D.C.); 15257080049@163.com (X.G.); 2 School of Chemical Engineering and Technology, Hebei University of Technology, Tianjin 300130, China

**Keywords:** phase change materials, wrinkled mesoporous silica, polyethylene glycol, thermal energy storage

## Abstract

Nanoflower-like wrinkled mesoporous silica (NFMS) was prepared for further application as the carrier of polyethylene glycol (PEG) to fabricate the new, shape-stabilized phase change composites (PEG/NFMS); NFMS could improve the loading content of PEG in the PEG/NFMS. To investigate the properties of PEG/NFMS, characterization approaches, such as scanning electron microscopy (SEM), transmission electron microscopy (TEM), Fourier transform infrared (FT-IR) spectroscopy, X-ray diffraction (XRD), thermal gravimetric analysis (TGA), Brunauer-Emmett-Teller (BET) analysis, and differential scanning calorimetry (DSC), were carried out. The characterization results illustrated that the PEG was completely adsorbed in the NFMS by physical adsorption, and the nanoflower-like wrinkled silica did not affect the crystal structure of PEG. As reported by the DSC test, although NFMS had a restriction influence on the activity of the PEG molecules, the melting and binding enthalpies of the PEG/NFMS could reach 136.6 J/g and 132.6 J/g, respectively. In addition, the TGA curves demonstrated that no evident weight loss was observed from 20 °C to 190 °C for the PEG/NFMS, and the results revealed that the PEG/NFMS had remarkable thermal stability. These results indicated that the NFMS is a potential carrier of organic phase change material for the preparation of shape-stabilized phase change composites.

## 1. Introduction

With the rapid development of the economy, the human demand for energy is also growing [1]. Sustainable and renewable energy has attracted much attention from investigators as the shortage of energy and the serious environmental problems restrict the social economy from developing healthily [1,2,3]. Phase change materials (PCMs), also known as latent heat storage materials, can absorb or release large amounts of heat and adjust the temperature of the surrounding environment during the phase transition process [1,4]. Because of their merits, phase change materials (PCMs) have been applied extensively in many fields, including heat exchangers, waste heat recovery, refrigeration, and air-conditioning systems [4,5,6,7]. PCMs can be classified into three categories: inorganic, organic and composite phase change materials. Compared with inorganic PCMs, organic PCMs include carboxylic acids, paraffins, and polyols, and have many significant merits, such as low price, low corrosion, small volume change, and supercooling [1,3,6,7]. As one of the common organic phase transition materials, polyethylene glycol (PEG) has the superiorities of constant phase transition temperature, thermal storage efficiency, non-toxicity, competitive price, low vapor pressure, and controlled melting point [6,8,9,10]. Therefore, it has attracted extensive attention in the study of phase change energy storage materials. However, the inevitable problem of PEG is that practical application issues, such as low thermal conductivity and leakage of solid–liquid PCMs limit its further use [9,11]. Effective methods were considered to resolve the above shortcomings; one of those is to fabricate form-stable phase change materials. In addition, silica, building materials, and some polymers have been used as the shape stabilization supports [3,9,12]. Mesoporous silica has numerous excellent advantages, including low cost, unique pore structures, ease of fabrication, high specific surface areas, and large pore volume [7,11], which means that mesoporous silica is a good supporting material. As a typical inorganic amorphous material, researchers have focused their great attention on mesoporous silica that was used for phase change material carriers. Min et al. prepared a novel type of organic PCMs by using radical mesoporous silica (RMS) sphere as the supporter and proved that radical mesoporous silica sphere was an ideal supporter in producing content-stable phase change materials [7]. Qian et al. reported that radial-like mesoporous silica sphere is able to promote the loading capacity of PEG, and the prepared shape-stabilized phase change material had fine mechanical strength, good thermal conductivity, and thermal stability [13]. Kadoono et al. used porous silica as the supporting material of lauric acid to prepare form-stable phase change composites, which could keep their capabilities for heat storage and release after many thermal cycles [14].

Recently, because the nanoflower-like wrinkled mesoporous silica (NFMS) has superior merits, including special pore structures, high absorption capacity, and large BET-specific areas, its application has attracted the concerns of increasing numbers of researchers, and the NFMS could be used widely in practical applications, such as drug delivery/release, separate functional food ingredients, and photoconductivity for electronics [7,15,16]. The NFMS has a radial mesoporous pore, which could facilitate the entrance of PEG molecules into its internal pores and improve the loading capacity. In addition, the nanoflower-like silica with wrinkled channels could be beneficial in preventing the leakage of the PEG. However, to the best of our knowledge, there have been no previous studies of nanoflower-like wrinkled mesoporous silica used as the carrier of phase change materials before now.

Thus, in this work, the nanoflower-like wrinkled mesoporous silica was synthesized first, and then the phase change materials (PEG/NFMS) were developed using the nanoflower-like wrinkled silica as the carrier of PEG. The PEG/NFMS was studied by differential scanning calorimetry (DSC), infrared spectroscopy (FTIR), thermal gravimetric analysis (TGA), X-ray diffraction (XRD), scanning electron microscopy (SEM), and transmission electron microscopy (TEM), and the results revealed that the PEG/NFMS has been proven to be a promising material for thermal energy storage applications.

## 2. Materials and Methods

### 2.1. Materials

Polyethylene glycol (PEG; Mw 4000 g/mol) and cyclohexane were obtained from Aladdin Industrial Corporation (Shanghai, China). Alcohol, n-butyl alcohol, tetraethyl orthosilicate (TEOS), cetyltrimethyl ammonium bromide (CTAB), and urea of analytical grade were purchased from Sinopharm Group Chemical Co, Ltd. (Shanghai, China). All of the chemicals were of analytical reagent grade and used as received without further purification.

### 2.2. Preparation of NFMS

For the preparation of the NFMS, polyethylene glycol and tetraethyl orthosilicate were used as the template and silica precursor, respectively. In a typical experiment, CTAB (1.0 g), n-butyl alcohol (1.0 g), aqueous urea solution (0.4 M, 30.0 g), and cyclohexane (12 mL) were put in a 100 mL round-bottom flask at ambient temperature with slow stirring for 5 min. Then TEOS (2.0 g) was added dropwise to the resulting solution with slow vibrating for 10 min. After constant stirring for 30 min at 25 °C, the reaction was continued at 70 °C for another 20 h. Then the products were collected by high-speed centrifugation (10,000 rpm) and tautologically washed with acetone and ethanol several times to remove the polyethylene glycol and unreacted TEOS. The precipitates were put in an incubator at 45 °C overnight. After calcining, NFMS was obtained.

### 2.3. Preparation of PEG/NFMS

In a typical synthetization, the vacuum impregnation technique was adopted to fabricate PEG/NFMS composites via impregnating PEG into NFMS’ porous network structure. First, PEG was dissolved into alcohol (15 mL) at room temperature in mass fractions of 60%, 70%, and 80%, and then NFMS (50 mg) was dispersed in the mixture. Secondly, the solution was stirred vigorously at approximately 600 r/min at 65 °C. Finally, the suspension was dried in an oven at 65 °C for 24 h. As a result, the PEG/NFMS shape-stabilized composites were obtained.

### 2.4. Characterization

The scanning electron microscopy (SEM, Quanta FEG-250, FEI Company, Waltham, MA, USA) images of NFMS and PEG/NFMS were observed at an accelerating voltage (200 kV). The micrographs of NFMS were obtained with transmission electron microscopy (TEM, JEM-2100F, JEOL, Tokyo, Japan). The crystal structures and crystallization properties of PEG and PEG/NFMS were determined by X-ray diffraction (XRD, D/MAX-2500, Rigaku, Akishima, Japan) at 40 kV and 30 mA from 10–60°. The thermogravimetric analyses (TGA, HCT-1, Henven Scientific Instument Factory, Beijing, China) of PEG and PEG/NFMS were measured in a high-purity flow of nitrogen, at which the heating rate is 10 °C/min between 20 and 500 °C. The chemical compatibilities of PEG, NFMS, and PEG/NFMS were characterized by infrared spectroscopy (FT-IR, MAGNA-IR 750, Nicolet Company, Waltham, MA, USA). The differential scanning calorimetry (DSC, 200-F3, NETZSCH, Munich, Germany) was used to analyze the thermal energy storage properties of PEG and PEG/NFMS. The tests to investigate the thermal behaviors were performed within a temperature range between 10 and 100 °C with a 10 °C/min rate, both in heating and cooling processes under the nitrogen flow. Nitrogen adsorption–desorption experiments were conducted on a Brunauer-Emmett-Teller instrument (BET, nova2000e, Quanta, Waltham, MA, USA), and the surface area, average pore size, and pore volume of the samples can be calculated with it.

## 3. Results and Discussion

### 3.1. Characterization of the NFMS and PEG/NFMS

**Figure 1:**
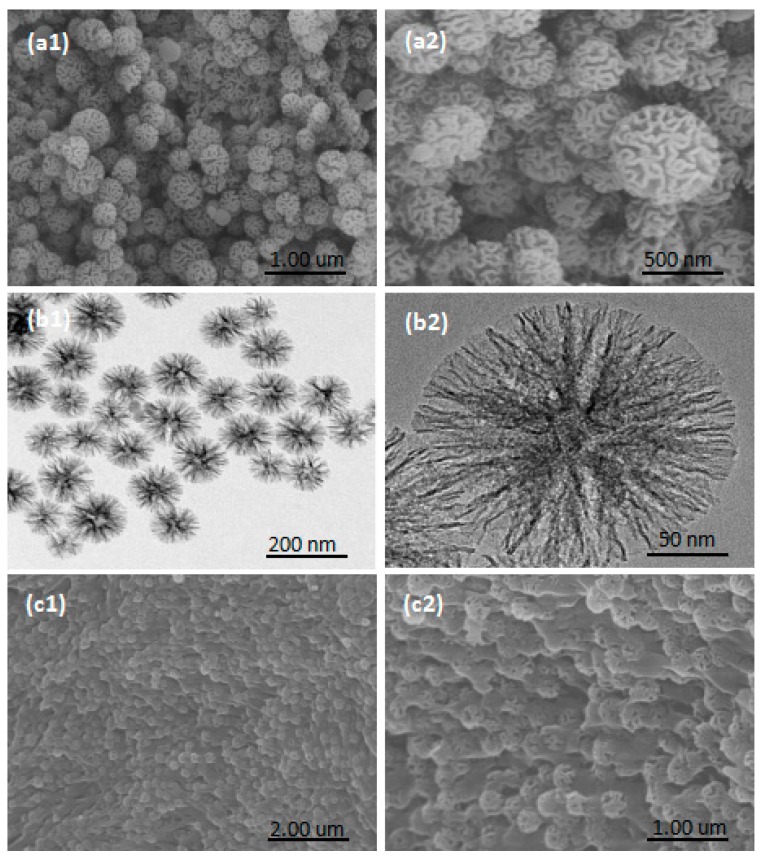
Scanning electron microscopy (SEM) (**a**) and transmission electron microscopy (TEM) (**b**) images of nanoflower-like wrinkled mesoporous silica (NFMS); SEM images (**c**) of Polyethylene glycol immobilized in NFMS (PEG/NFMS).

Figure 1a,b presents the SEM and TEM images of the NFMS. According to the SEM image (Figure 1a), NFMS nanoparticles were made up of spherical particles with wrinkles of various diameters and evident flower-like morphology with rough surfaces. TEM images (Figure 1b) showed that NFMS nanoparticles were arranged in relatively neat snowflake nanocrystals, and the diameter of the particles ranged from about 90–180 nm. The SEM images of PEG/NFMS composites are shown in Figure 1c, and it could be seen that PEG was well distributed in composite materials due to the effect of surface tension, as well as hydrogen bonding between the PEG molecules and the hydroxy on the surface of NFMS [17,18]. Moreover, NFMS composites provided great BET surface areas, high porosity, and large pore volume for the whole composites, which contributed to absorption between the PEG and the matrix and prevented the leakage of the melted PEG. 

**Figure 2:**
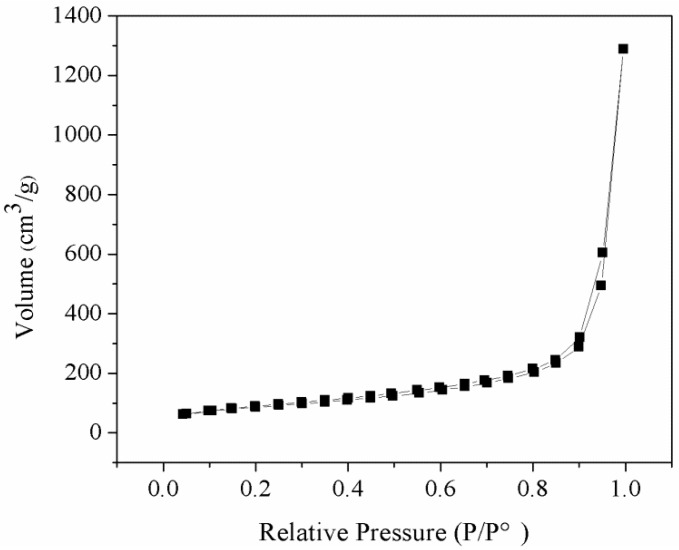
Nitrogen adsorption–desorption isotherm.

Figure 2 illustrates the nitrogen adsorption–desorption isotherm of the NFMS, which exhibited the type IV isotherm, which had an adsorption hysteresis loop at relative pressure p/p^o^ of 0.9–1.0. Therefore, the isotherm of the NFMS could be classified as type IV isotherm, which was consistent with the mesoporous material [19,20]. The specific surface area of the NFMS was 296.1 m^2^/g according to the BET method, and its total pore volume and average pore size were calculated to be 1.99 cm^3^/g and 2.64 nm, respectively, according to the Barrett-Joyner-Halenda (BJH) method. The large pore volume and pore size of the NFMS were favorable for the PEG molecules to enter into the inner pores of the NFMS.

### 3.2. Crystallization Properties of PEG, NFMS, and PEG/NFMS 

**Figure 3:**
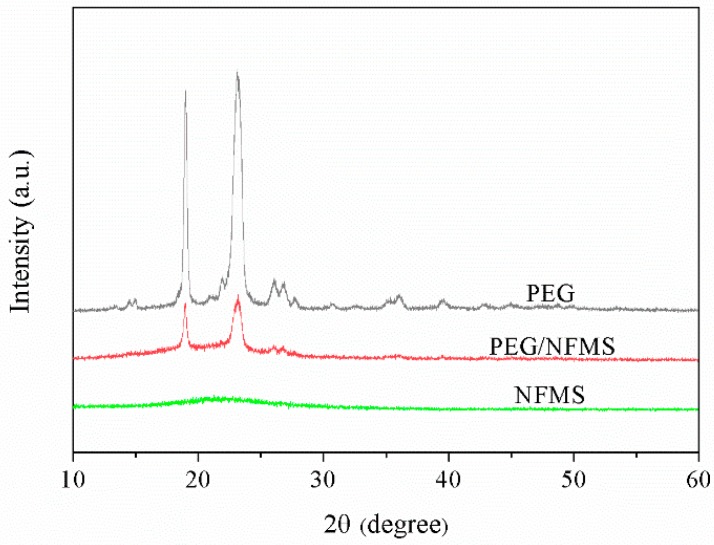
X-ray diffraction (XRD) patterns of PEG, NFMS, and PEG/NFMS.

Figure 3 depicts the wide-angle temperature-dependent XRD measurement results of pure PEG, NFMS, and PEG/NFMS between 10° and 60°. It could be observed that there was only a broad band between 18 and 30° in the pattern of NFMS, and no explicit peaks occurred, suggesting that the NFMS owned a typical non-crystalline structure [18]. The peaks at approximately 19°, 23°, 26°, and 37° in the wave pattern of the PEG belonged to PEG crystal [17]. For PEG/NFMS, the conspicuous high diffraction peaks of PEG appeared. This phenomenon indicated that NFMS successfully adsorbed PEG, and did not destroy the crystal structure of PEG [21]. Furthermore, chemical interaction did not occur between PEG and NFMS because no new peaks appeared.

### 3.3. Chemical Properties of Pure PEG, NFMS, and PEG/NFMS 

**Figure 4:**
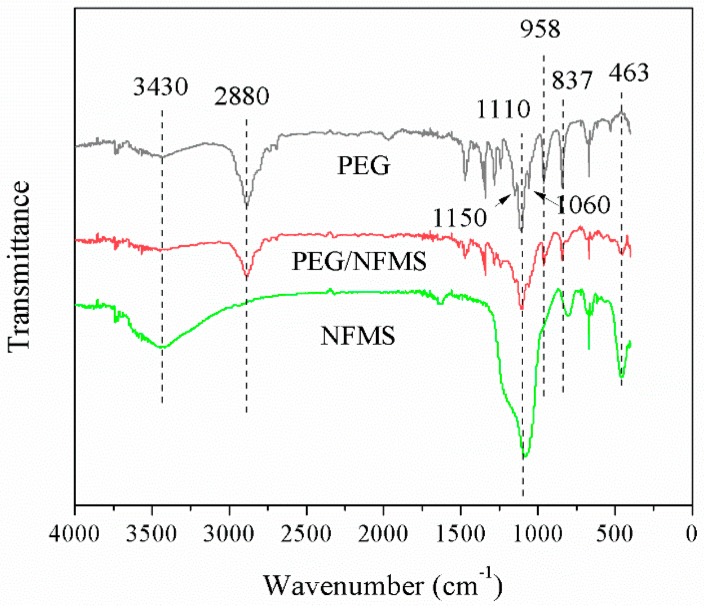
Fourier transform infrared (FT-IR) spectra of pure PEG, NFMS, and 80% PEG/NFMS.

The FT-IR spectra of the pure PEG, NFMS, and PEG/NFMS are shown in Figure 4. The band located at 3430 cm^−1^ for pure PEG was an asymmetric stretching vibration of the functional group of O–H [5,22]. The characteristic absorption peaks at 958 cm^−1^ and 2880 cm^−1^ should represent the groups of C-H and -CH_2_, respectively [17]. At the same time, the triplet peaks of the C-O-C groups at 1150 cm^−1^, 1110 cm^−1^ and 1060 cm^−1^ could be observed [5]. For the NFMS, the bands at 463 cm^−1^, 837 cm^−1^, and 1110 cm^−1^ were consistent with symmetric stretching vibrations, which were caused by Si-O-Si groups [7]. For PEG/NFMS, the main peaks overlapped with the peaks of the PEG and the NFMS, accompanied by some slight shifts. No other obvious new peaks could be observed, which confirmed that there were only physical interactions between the PEG molecules and the NFMS, including surface tension and capillary [20].

### 3.4. Leakage Test of PEG/NFMS 

**Figure 5:**
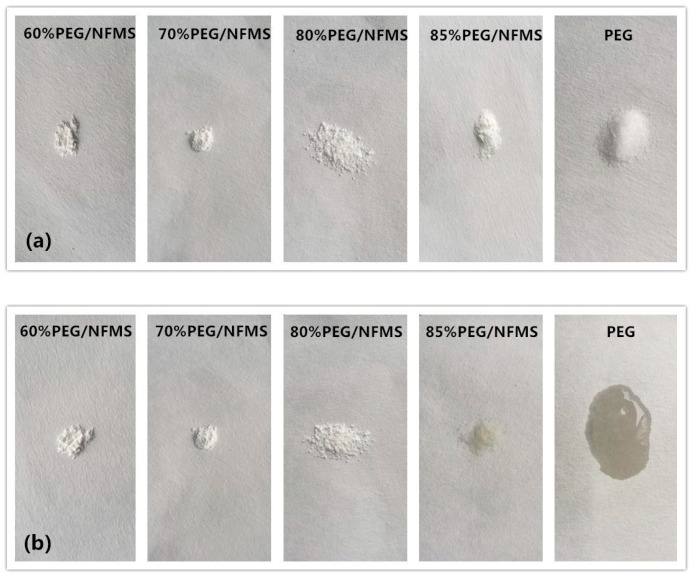

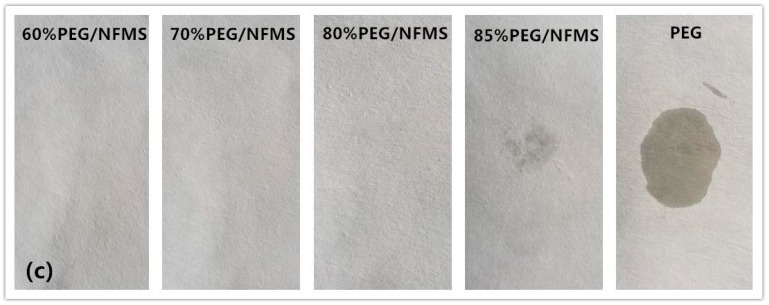
Photographs of leakage test of PEG/NFMS: (**a**) before the thermal stability test; (**b**) after the thermal stability test; (**c**) after removal of the samples.

To confirm whether the samples would leak in a high temperature environment, a thermal stability test of the innocent material of PEG and the composite PCMs was implemented in an incubator at 75 °C for 15 min. In this experiment, one pure PEG sample and three composite material samples were tested, and the results were presented in Figure 5. Figure 5a,b presents the photographs before and after the thermal stability test, and Figure 5c shows the photographs after removing the samples. The composite materials were synthesized with mesoporous silica and the PEG in different proportions, in which the content of PEG was 60%, 70%, 80%, and 85%, respectively. As shown in Figure 5, no leakage of 60%, 70%, and 80% composites was observed. However, the 85% PEG/NFMS was observed at a slight leakage in the experiments. Specifically, the pure PEG melted completely because the temperature of the incubator exceeds its melting point of 58 °C [23]. However, even the 80% PEG/NFMS composites experienced heating–cooling cycles 50 times, and no leakage has been found. This is because the PEG molecules were fixed effectively to the external and internal of NFMS. The large specific surface area and mesoporous network of the NFMS restrained the leakage of the PEG molecules. The leakage test showed that NFMS has a positive effect on the immobilization of the PEG. The experimental results finally indicated that with the proportion of the PEG becoming higher, the ability of mesoporous silica to prevent the leakage of the PEG molecules was diminished [24]. From what was discussed above, 80% of PEG was truly the best concentration for the preparation of PEG/NFMS.

### 3.5. Thermal Properties of PEG and PEG/NFMS 

**Figure 6:**
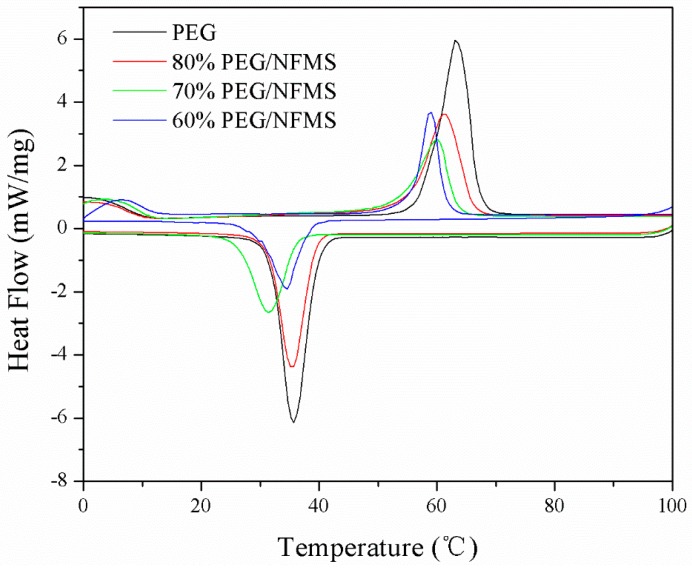
Differential scanning calorimetry (DSC) curves of PEG and PEG/NFMS at different concentrations.

The DSC analysis technique was applied to evaluate the fusion and crystallization enthalpies of the PEG and PEG/NFMS. In Figure 6 and Table 1, the DSC curves of the PEG and the PEG/NFMS PCMs with PEG contents ranging from 60–80% and the relevant data are shown. As shown in Table 1, the melting and solidifying enthalpies of pristine PEG were 202.1 J/g and 186.4 J/g, respectively. According to Figure 6, the endothermic peak of pristine PEG was at 63.1 °C and the exothermic peak was at 35.7 °C. Compared with the pure PEG, the enthalpy values of the PEG/NFMS with varied mass ratio had decreased significantly, which further confirmed that the PEG crystallization had intervened [25,26]. It was obvious that the enthalpies of PEG/NFMS, which were different from each other, increased while the PEG content increased accordingly. The enthalpy was very small, as the PEG content was lower than 60%. The melting and solidifying enthalpies of 80% PEG/NFMS could reach 136.6 J/g and 132.6 J/g, respectively. According to Table 2, 80% PEG/NFMS was compared with other PCMs that were reported in the references, and the results showed that the PEG/NFMS had competitive fusion and solidification enthalpies and had great potential for energy storage in practical applications [27].

### 3.6. Thermal Stability of Pure PEG and 80 w% PEG/NFMS 

**Figure 7:**
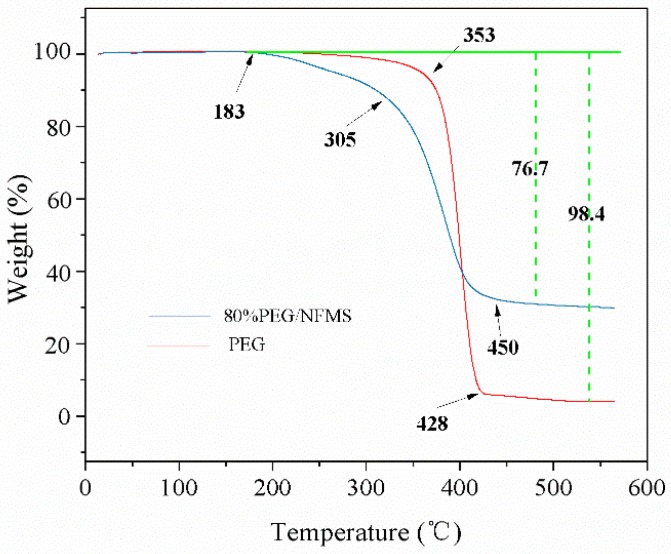
Thermal gravimetric analysis (TGA) curves of pure PEG and 80 w% PEG/NFMS.

The thermogravimetric analysis (TGA) curves of PEG and PEG/NFMS composites are presented in Figure 7. The PEG and PEG/NFMS only experienced one sharp stage of weight loss process due to their purity for both PEG and PEG/NFMS, and degraded at temperatures of 183 °C. For pristine PEG, slight weight loss started to appear at 183 °C, and then sharp weight loss took place from 353–428 °C (which could be explained by the decomposition of the PEG chains), and approximately 1.6% residual substances remained in it. In regard to the PEG/NFMS, there was no distinct decomposition reaction before 305 °C. After this temperature range, the composite evaporated rapidly, until evaporating completely at 450 °C, which illustrated that when the temperature was below 183 °C, the composite had a good thermal stability [30]. According to the final result, the content of the PEG in the PEG/NFMS was 76.7%; therefore, the actual content of the PEG in the PEG/NFMS was lower than the designed amount in the fabrication process, which could be attributed to a small amount of PEG molecules being splashed on the wall of the conical flask in the synthesis process. In addition, the TGA test confirmed that the PEG molecules were stably fixed in the pores of NFMS.

### 3.7. Undercooling of PEG and NFMS

**Figure 8:**
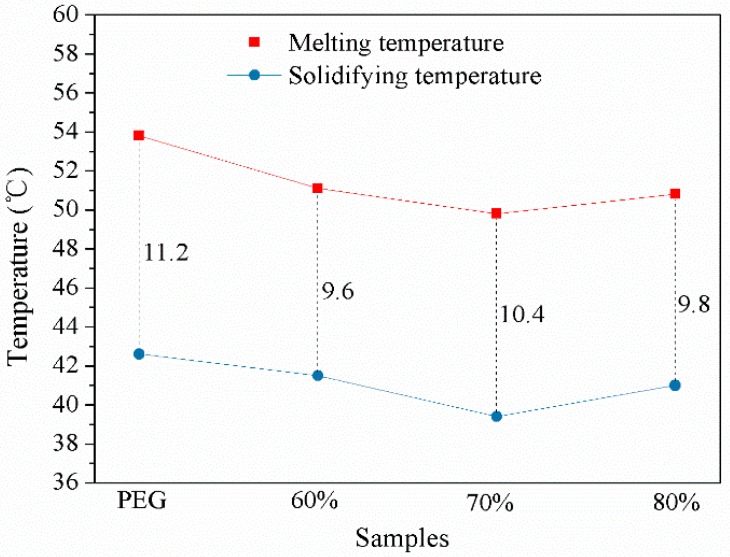
The extent of undercooling of PEG and PEG/NFMS.

In practical applications, undercooling of the PEG and PEG/NFMS, which is shown in Figure 8, is an important parameter. The evaluation tests revealed that the extent of undercooling of the PEG/NFMS composites was lower than that of the pristine PEG. This was attributed to the fact that when the PEG was blended with the mesoporous NFMS, the extent of undercooling of the PEG could profitably decline. This suggested that the prepared PCMs had good potential for practical applications in thermal energy storage.

### 3.8. Percentage of Heat Loss

**Figure 9:**
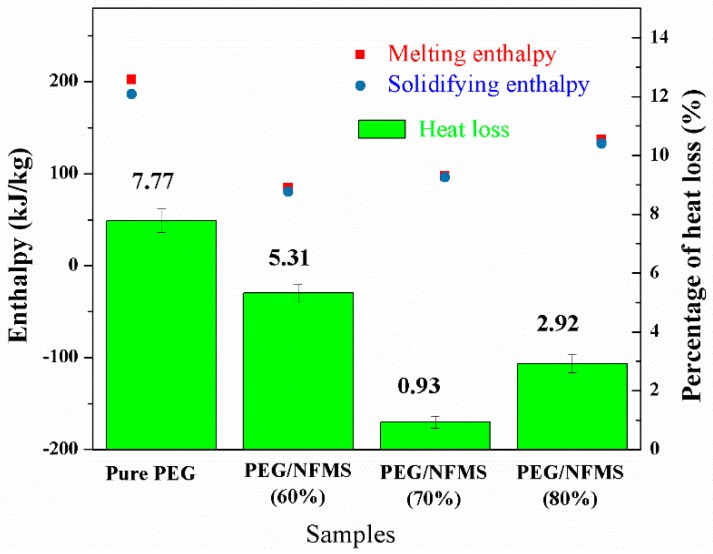
Comparisons of phase change enthalpy and heat loss percentage for pure PEG and PEG/NFMS.

In Figure 9, the phase change enthalpy and heat loss percentage for pristine PEG and the PEG/NFMS are shown. As depicted in Figure 9, the melting and solidifying enthalpies of the PEG/NFMS were lower than that of the pure PEG, and the 80% PEG/NFMS had the highest enthalpy in different proportions. In comparison with the pristine PEG, the heat loss percentage of the PEG/NFMS composites with different mass ratios of PEG decreased due to the blending of mesoporous silica; therefore, the heat storage property of the PEG/NFMS composites was increased. 

## 4. Conclusions

In this study, a form-stable phase change composite of PEG/NFMS was successfully fabricated by using nanoflower-like wrinkled mesoporous silica as the matrix of polyethylene glycol. The characteristics of PEG/NFMS were studied via SEM, BET, TEM, FT-IR, XRD, TGA, and DSC. The DSC measurements demonstrated that when the PEG content was 80%, the melting and solidification enthalpies of the PEG/NFMS composites were 136.8 J/g and 132.8 J/g, respectively. The TGA result showed that the actual maximum content of PEG was 76.7%. It was revealed that the PEG molecules were stably fixed in the PEG/NFMS with no melted PEG leaking out, and there was only a physical absorption process between the PEG and NFMS. In conclusion, the PEG/NFMS composite with high heat storage properties has been proven to be a promising candidate for widespread heat storage applications.

## Figures and Tables

**Table 1:** Thermal characteristics of polyethylene glycol (PEG) and polyethylene glycol immobilized in nanoflower-like wrinkled mesoporous silica (PEG/NFMS).

Samples	PEG Content	Melting			Crystallization		
		T_mp_ (°C )	T_m_ (°C )	H_m_ (J/g)	T_cp_ (°C )	T_c_ (°C )	H_c_ (J/g)
PEG	100%	63.1	53.8	202.1	35.7	42.6	186.4
PEG/NFMS	60%	59.1	51.1	84.7	34.5	41.5	80.2
PEG/NFMS	70%	60.0	49.8	97.0	31.4	39.4	96.1
PEG/NFMS	80%	60.9	50.8	136.8	35.2	41.0	132.8

**Table 2:** Thermal characteristics of different composite phase change materials (PCMs) in the literature.

PCMs	PEG	Melting		Crystallization		Reference
	Content	T_m_ (°C)	H_m_ (J/g)	T_c_ (°C)	H_c_ (J/g)	
PEG/NFMS	80%	50.80	136.60	41.00	132.60	Present study
SA/TAMSN	70%	71.50	108.80	64.00	114.10	Chen et al. [1]
PEG/RMS	80%	57.22	129.60	39.02	118.30	Min et al. [7]
PEG6000/CNIC	60%	43.80	45.80	12.00	42.70	Feng et al. [9]
PEG/AC	80%	49.00	81.30	27.80	72.80	Feng et al. [11]
PEG/SiO_2_	79.3%	58.09	151.80	42.34	141.00	Qian et al. [28]
c-PCMP4	85.36%	56.50	159.70	37.90	155.60	Zhao et al. [29]

## References

[1] Chen Y., Zhang X.J., Wang B.F., Lv M.J., Zhu Y.Y., Gao J.K. (2017). Fabrication and characterization of novel shape-stabilized stearic acid composite phase change materials with tannic-acid-templated mesoporous silica nanoparticles for thermal energy storage. R. Soc. Chem..

[2] Gao J., Kong W.X., Zhou L.Y., He Y., Ma L., Wang Y., Yin L.Y., Jiang Y.J. (2017). Monodisperse core-shell magnetic organosilica nanoflowers with radial wrinkle for lipase immobilization. Chem. Eng. J..

[3] Goitandia A.M., Beobide G., Aranzabe E., Aranzabe A. (2015). Development of content-stable phase change composites by infiltration into inorganic porous supports. Sol. Energy Mater. Sol. Cells.

[4] Cai Y.B., Sun G.Y., Liu M.M., Zhang J., Wang Q.Q., Wei Q.F. (2015). Fabrication and characterization of capric-lauric-palmitic acid/electrospun SiO_2_, nanofibers composite as form-stable phase change material for thermal energy storage/retrieval. Sol. Energy.

[5] Li J.R., He L.H., Liu T.Z., Gao X.J., Zhu H.Z. (2013). Preparation and characterization of PEG/SiO_2_ composites as shape-stabilized phase change materials for thermal energy storage. Sol. Energy Mater. Sol. Cells.

[6] Wang J.J., Yang M., Lu Y.F., Jin Z.K., Tan L., Gao H.Y., Fan W.J., Wang G. (2016). Surface functionalization engineering driven crystallization behavior of polyethylene glycol confined in mesoporous silica for shape-stabilized phase change materials. Nano Energy.

[7] Min X., Fang M.H., Huang Z.H., Liu Y.G., Huang Y.T., Wen R.L., Qian T.T., Wu X.W. (2015). Enhanced thermal properties of novel shape-stabilized PEG composite phase change materials with radial mesoporous silica sphere for thermal energy storage. Sci. Rep.-UK.

[8] Gao J.K., Hou L.A., Zhang G.H., Gu P. (2015). Facile functionalized of SBA-15 via a biomimetic coating and its application in efficient removal of uranium ions from aqueous solution. J. Hazard. Mater..

[9] Feng L.L., Song P., Yan S.C., Wang H.B., Wang J. (2015). The shape-stabilized phase change materials composed of polyethylene glycol and graphitic carbon nitride matrices. Thermochim. Acta.

[10] Qi L.M. (2010). Colloidal chemical approaches to inorganic microand nanostructures with controlled morphologies and patterns. Coord. Chem. Rev..

[11] Feng L.L., Zhao W., Zheng J., Frisco S., Song P., Li X.G. (2011). The shape-stabilized phase change materials composed of polyethylene glycol and various mesoporous matrices (AC, SBA-15 and MCM-41). Sol. Energy Mater. Sol. Cells.

[12] Moon D.S., Lee J.K. (2012). Tunable Synthesis of Hierarchical Mesoporous Silica Nanoparticles with Radial Wrinkle Structure. Langmuir.

[13] Qian T.T., Li J.H., Min X., Deng Y., Guan W.M., Ning L. (2016). Radial-like mesoporous silica sphere: A promising new candidate of supporting material for storage of low-, middle-, and high-temperature heat. Energy.

[14] Kadoono T., Ogura M. (2014). Heat storage properties of organic phase-change materials confined in the nanospace of mesoporous SBA-15 and CMK-3. Phys. Chem. Chem. Phys..

[15] Ryan B., Brad W., Michellel G.L., Andrea D.J. (2008). Hierarchical mesoporous silica materials for separation of functional food ingredients-A review. Innov. Food. Sci. Emerg..

[16] Wang Y., Herron N. (1996). X-Ray Photoconductive Nanocomposites. Science.

[17] Chen Y., Zhu Y.Y., Wang J.B., Lv M.J., Zhang X.J., Gao J.K., Zhang Z.J., Lei H. (2017). Novel Shape-Stabilized Phase Change Materials Composed of Polyethylene Glycol/Nonsurfactant-Templated Mesoporous Silica: Preparation and Thermal Properties. JOM-UK.

[18] Gao J.K., Lv M.J., Lu J.S., Chen Y., Zhang Z.J., Zhang X.J., Zhu Y.Y. (2017). Enhanced Thermal Properties of Novel Latent Heat Thermal Storage Material Through Confinement of Stearic Acid in Meso-Structured Onion-Like Silica. JOM-US.

[19] Moon D.S., Lee J.K. (2014). Formation of wrinkled silica mesostructures based on the phase behavior of pseudoternary systems. Langmuir.

[20] Deng Y., Li J.H., Nian H.G., Li Y.L., Yin X.P. (2017). Design and preparation of shape-stabilized composite phase change material with high thermal reliability via encapsulating polyethylene glycol into flower-like TiO_2_, nanostructure for thermal energy storage. Appl. Therm. Eng..

[21] Abu-Zied B.M., Hussein M.A., Asiri A.M. (2015). Characterization, in situ electrical conductivity and thermal behavior of immobilized PEG on MCM-41. Int. J. Electrochem. Sci..

[22] Golestaneh S.I., Mosallanejad A., Karimi G., Khorram M., Khashi M. (2016). Fabrication and characterization of phase change material composite fibers with wide phase-transition temperature range by co-electrospinning method. Appl. Energy.

[23] Malach T.J. (2002). Phase Change Formulation. U.S. Patent.

[24] Sarı A., Karaipekli A. (2008). Preparation, thermal properties and thermal reliability of capric acid/expanded perlite composite for thermal energy storage. Mater. Chem. Phys..

[25] Ke H.Z., Li D.W., Zhang H.D., Wang X.L., Cai Y.B., Huang F.L., Wei Q.F. (2013). Electrospun form-stable phase change composite nanofibers consisting of capric acid-based binary fatty acid eutectics and polyethylene terephthalate. FIB Polym..

[26] Wang C.L., Yeh K.L., Chen C.W., Lee Y., Lee H.-L., Lee T. (2017). A quick-fix design of phase change material by particle blending and spherical agglomeration. Appl. Energy.

[27] Cai Y.B., Ke H.Z., Lin L., Fei X.Z., Wei Q.F., Song L., Hu Y., Fong H. (2012). Preparation, morphology and thermal properties of electrospun fatty acid eutectics/polyethylene terephthalate form-stable phase change ultrafine composite fibers for thermal energy storage. Energy Convers. Manag..

[28] Qian T.T., Li J.H., Ma H.W., Yang J. (2015). The preparation of a green shape-stabilized composite phase change material of polyethylene glycol/SiO_2_, with enhanced thermal performance based on oil shale ash via temperature-assisted sol–gel method. Sol. Energy Mater. Sol. Cells.

[29] Zhao Y.J., Min X., Huang Z.H., Fang M.H. (2017). Honeycomb-like structured biological porous carbon encapsulating PEG: A shape-stable phase change material with enhanced thermal conductivity for thermal energy storage. Energy Build..

[30] Pan L., Tao Q.H., Zhang S.D., Wang S.S., Zhang J., Wang S.H., Wang Z.Y., Zhang Z.P. (2012). Preparation, characterization and thermal properties of micro-encapsulated phase change materials. Sol. Energy Mater. Sol. Cells.

